# Sodium-glucose cotransporter-2 inhibition for heart failure with preserved ejection fraction and chronic kidney disease with or without type 2 diabetes mellitus: a narrative review

**DOI:** 10.1186/s12933-023-02023-y

**Published:** 2023-11-16

**Authors:** Robert J. Mentz, Stephen A. Brunton, Janani Rangaswami

**Affiliations:** 1 Division of Cardiology, Department of Medicine, Duke Clinical Research Institute, Duke University Medical Center, 40 Duke Medicine Circle, Durham, NC 27710 USA; 2 Primary Care Metabolic Group, Winnsboro, SC USA; 3 Division of Nephrology, George Washington University School of Medicine and Health Sciences, Washington, DC USA

**Keywords:** Cardio-kidney metabolic, Cardiorenal syndrome, Cardiovascular disease, Chronic kidney disease, Diabetic kidney disease, Heart failure with preserved ejection fraction, Sodium-glucose cotransporter-2 inhibitors, Type 2 diabetes mellitus

## Abstract

**Background:**

Heart failure (HF), chronic kidney disease (CKD), and type 2 diabetes mellitus (T2DM) are common and interrelated conditions, each with a significant burden of disease. HF and kidney disease progress through pathophysiologic pathways that culminate in end-stage disease, for which T2DM is a major risk factor. Intervention within these pathways can disrupt disease processes and improve patient outcomes. Sodium-glucose cotransporter-2 inhibitors (SGLT2is) have been investigated in patient populations with combinations of T2DM, CKD, and/or HF. However, until recently, the effect of these agents in patients with HF with preserved ejection fraction (HFpEF) was not well studied.

**Main body:**

The aim of this review is to summarize key information regarding the interaction between HFpEF, CKD, and T2DM and discuss the role of SGLT2 inhibition in the management of patients with comorbid HFpEF and CKD, with or without T2DM. Literature was retrieved using Boolean searches for English-language articles in PubMed and Google Scholar and included terms related to SGLT2is, HFpEF, T2DM, and CKD. The reference lists from retrieved articles were also considered.

**Conclusion:**

SGLT2is are efficacious and safe in treating HFpEF in patients with comorbid CKD with and without T2DM. The totality of evidence from clinical trials data suggests there are benefits in using SGLT2is across the spectrum of left ventricular ejection fractions, but there may be a potential for different renal effects in the different ejection fraction groups. Further analysis of these clinical trials has highlighted the need to obtain more accurate phenotypes for patients with HF and CKD to better determine which patients might respond to guideline-directed medical therapies, including SGLT2is.

**Graphical Abstract:**

*CI* confidence interval, *EF* ejection fraction, *eGFR* estimated glomerular filtration rate, *HF* heart failure, *HHF* hospitalization for HF, *HR* hazard ratio, *LVEF* left ventricular ejection fraction, *SGLT2i* sodium-glucose cotransporter-2 inhibitor, *UACR* urine albumin-creatinine ratio. **a** Mean value, unless otherwise stated, **b** SGLT2i vs. placebo, **c** Data reanalyzed using more conventional endpoints (≥ 50% sustained decrease in eGFR, and including renal death) (UACR at baseline not stated in trial reports)
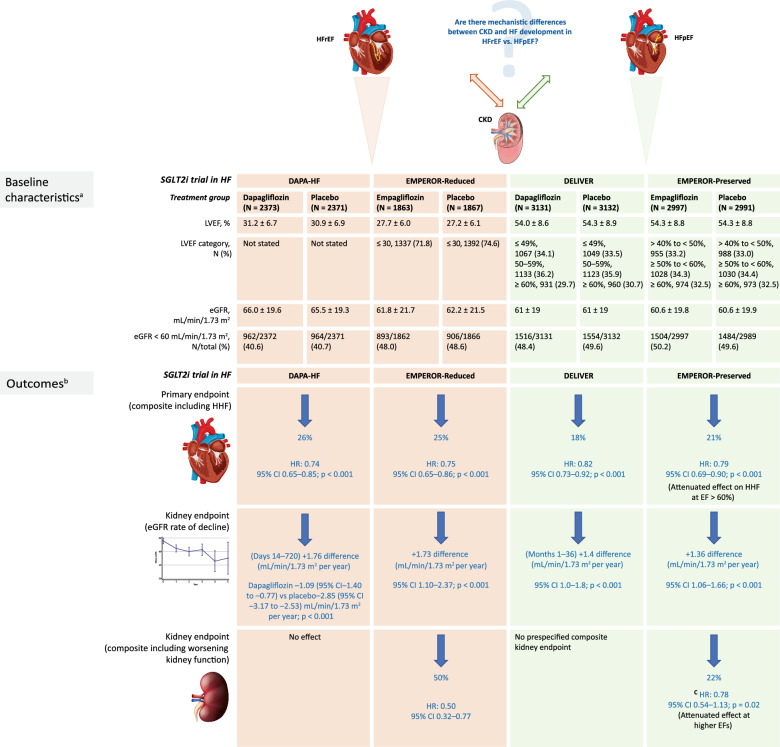

## Introduction

Heart failure (HF), chronic kidney disease (CKD), and type 2 diabetes mellitus (T2DM) are common and interrelated conditions, each conferring increased disease burden both individually and in combination [1]. Cardiovascular disease (CVD), particularly HF, and CKD each progress through a pathway of pathophysiologic steps, for which T2DM is a major risk factor [2]. Sodium-glucose cotransporter-2 inhibitors (SGLT2is) are used to treat hyperglycemia in patients with T2DM. Data from large, phase 3, randomized controlled trials (RCTs) have shown that SGLT2i therapy improved cardiovascular (CV) and kidney outcomes in patients with T2DM [3], and observed reductions in the risk of hospitalization for HF led to this drug class being evaluated in patients with HF, with or without T2DM. SGLT2is have been shown to reduce the development and progression of HF with reduced ejection fraction (HFrEF) [4, 5]; however, until recently, the effect of these agents in patients with HF with preserved ejection fraction (HFpEF) was not well studied. The aim of this review is to summarize key information regarding the interaction between HFpEF, CKD, and T2DM and discuss the role of SGLT2 inhibition in the management of patients with comorbid HFpEF and CKD, with or without T2DM.

Literature was retrieved from PubMed and Google Scholar databases using Boolean searches for terms related to SGLT2is, HFpEF, T2DM, and CKD (limits: English-language articles, humans). The reference lists from retrieved articles were also considered. Other relevant literature was obtained on the basis of the personal knowledge and experience of the authors. Additional data were obtained from the US National Institutes of Health website ClinicalTrials.gov and from websites pertaining to individual therapeutic agents of interest. The retrieved references were manually assessed by one reviewer and formed the basis for this narrative review.

## Epidemiology of HF, CKD, and T2DM

### HF epidemiology

HF is defined as a clinical syndrome with symptoms and/or signs caused by a structural and/or functional cardiac abnormality and corroborated by elevated natriuretic peptide levels and/or objective evidence of pulmonary or systemic congestion [6]. Left ventricular ejection fraction (LVEF) provides prognostic information for patients with HF and defines differing treatment groups [6–8]: HF with reduced EF (HFrEF; LVEF ≤ 40%), previously called “systolic HF”; HF with mildly reduced EF (HFmrEF; LVEF 41–49%); HF with preserved EF (HFpEF; LVEF ≥ 50%), previously called “diastolic HF”; and HF with improved EF (baseline LVEF ≤ 40%, followed by a ≥ 10-point increase from baseline and a second LVEF measurement > 40%) [6]. HF affects approximately 64 million adults globally (per 2017 data) [9]. This represents an almost doubling in HF cases over a 27-year period (1990–2017), with almost half of all cases coming from China and India [9]. In the United States (US), HF prevalence was 2.4% in 2012 and affected 5.7 million adults (aged ≥ 20 years) and is expected to rise to 3.0% by 2030, when it will affect > 8 million adults (aged ≥ 20 years) [10]. In the community setting, up to half of patients with HF have HFpEF [11–13], although this rate depends on diagnostic accuracy and an evolving clinical definition [12]. Factors contributing to the increased prevalence of HFpEF include an aging population and increased HFpEF-related risk factors (such as diabetes, hypertension, and obesity), as well as improved diagnosis and survival [12]. Trial-based analyses, mainly involving patients with chronic HF in ambulatory settings (i.e., outpatient care), report 1-year mortality rates for HFpEF of around 5%, whereas observational studies report rates of up to 30% using data primarily from inpatients with decompensated HFpEF [12]. The 5-year mortality rate in a community study of adults with HFpEF (aged > 45 years) was 10% for those with a mild degree of diastolic dysfunction, rising to 23% in moderate/severe disease [14]. A large meta-analysis of ambulatory patients with HF found that pooled survival rates were similar for HFpEF and HFrEF at 1 year (89% and 88%, respectively) and 5 years (70% and 63%, respectively) [15].

### CKD epidemiology

CKD is defined as persistent albuminuria (albumin-creatinine ratio [ACR], ≥ 30 mg/g [≥ 3 mg/mmol]), persistently reduced estimated glomerular filtration rate (eGFR; < 60 mL/min per 1.73 m^2^), or both [16]. The Global Burden of Disease Study (2017 data) reported 698 million cases of all-stage CKD, giving a global prevalence of 9.1% [17], whereas an earlier systematic review and meta-analysis (100 studies; ~ 6,900,000 patients) reported a global prevalence of 13.4% [18]. In the US, the prevalence of CKD was 15% in 2021, equating to approximately 37 million American adults with CKD [19]. Changes in CKD prevalence over time have showed stabilization or even improvement (reviewed in [20]). The reasons for this are unclear, given the observed increases in CKD risk factors (such as T2DM and obesity), although hypertension prevalence has stabilized or decreased in many high- and middle-income countries due to improved detection and treatment [21]. The primary causes of CKD are T2DM (30–50% of cases), hypertension (~ 27%), and primary glomerulonephritis (~ 8%) [22]. The prognosis of CKD worsens with increasing Kidney Disease: Improving Global Outcomes (KDIGO) CKD category (based on GFR and albuminuria categories) [23, 24], but only a small proportion of individuals have severely decreased GFR (stage G4), kidney failure (G5), or severely increased albuminuria (A3) [23, 24]. In addition to the complications associated with CKD (such as anemia, mineral bone disease, end-stage kidney disease [ESKD], etc.), CKD is an important risk factor for CV morbidity and mortality, including coronary artery disease, HF, arrhythmias, and sudden CV death (reviewed in [25]). For individuals with CKD, the risk of developing CVD is greater than that of developing ESKD [25, 26].

### T2DM epidemiology

T2DM accounts for up to 95% of all cases of diabetes, and is caused by a progressive loss of insulin secretion from pancreatic β-cells that becomes insufficient to compensate for insulin resistance, resulting in hyperglycemia [27]. T2DM can be diagnosed using various tests (e.g., fasting plasma glucose, glycated hemoglobin, etc.) and in a variety of clinical settings (e.g., incidental finding, asymptomatic/symptomatic screening, etc.) [27]. Traditional risk factors for T2DM include overweight and obesity, lack of physical activity, and unhealthy diet. Around 6% of the global population (~ 462 million people) are affected by T2DM, and T2DM accounted for more than 1 million deaths (Global Burden of Disease data from 2017) [28]. In the US, 35.4 million adults have T2DM (2019 data) [29]. In terms of disability-adjusted life years (DALYs), a measure of premature deaths and years lived with disability from a particular disease, T2DM causes the seventh highest burden of disease [28]. Global prevalence of T2DM has increased over the last 30 years and is forecasted to rise (cases per 100,000 people) from 6059 in 2017 to 7079 by 2030 [28]; furthermore, global trends modeled to 2025 show continued increases in T2DM incidence, age-standardized rates, deaths, and DALYs [30]. It is well established that diabetes-induced hyperglycemia is a causative factor in the development of microvascular disease (retinopathy, nephropathy, and neuropathy) and macrovascular disease (peripheral artery disease, coronary artery disease, and stroke) [31–34], via the activation of pathways that trigger cellular oxidative stress, release of inflammatory mediators, mitochondrial dysfunction, and the development of atherosclerosis (reviewed in [35]). Atherosclerotic CVD (ASCVD) is the leading cause of morbidity and mortality in patients with T2DM, and conditions that commonly coexist with T2DM (such as hypertension and dyslipidemia) are risk factors for ASCVD, as is T2DM itself [36].

## The relationship between CKD and HF: cardiorenal syndrome

**Fig. 1:**
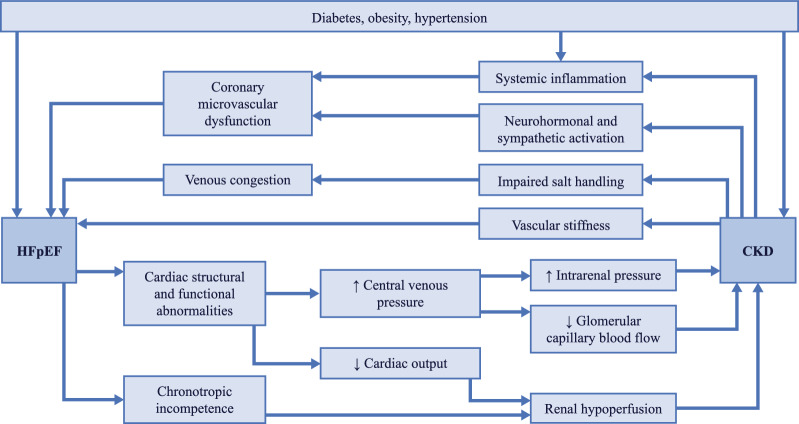
Summary of pathophysiological processes linking HFpEF and CKD [41]. HFpEF and CKD share common risk factors, including diabetes, hypertension, and obesity, but a number of pathophysiological processes also contribute to the interplay between cardiac and renal dysfunction. *CKD* chronic kidney disease, *HFpEF* heart failure with preserved ejection fraction. Reproduced from Joslin JR, Lioudaki E, Androulakis E. Interrelation between heart failure with preserved ejection fraction and renal impairment. Rev Cardiovasc Med. 2022;23(2):69. http://doi.org/10.31083/j.rcm2302069, by IMR Press

**Fig. 2:**
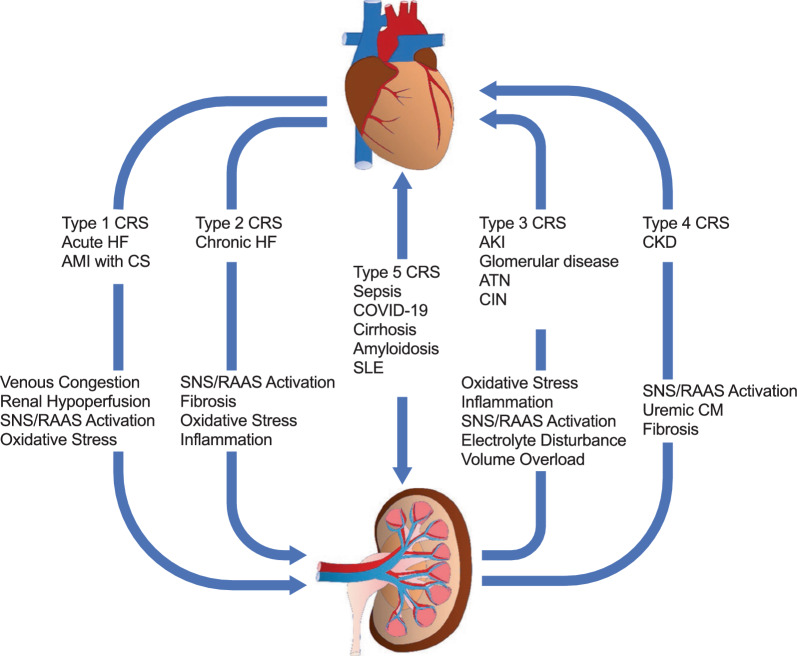
Classification of CRS [51]. *AKI* Acute kidney injury, *AMI* Acute myocardial infarction, *ATN* acute tubular necrosis, *CIN* contrast-induced nephropathy, *CKD* chronic kidney disease, *CM* cardiomyopathy, *COVID-19* coronavirus disease 2019, *CRS* cardiorenal syndrome, *CS* cardiogenic shock, *HF* heart failure, *RAAS* renin–angiotensin–aldosterone system, *SLE* systemic lupus erythematosus, *SNS* sympathetic nervous system. Reproduced from Curr Probl Cardiol. 2023;48(3), Kim JA, Wu L, Rodriguez M, et al. Recent developments in the evaluation and management of cardiorenal syndrome: a comprehensive review, page 101,509

HF and CKD have a bidirectional relationship [36], and the presence of either condition is associated with a worse prognosis in the other. Patients with CKD have a three-fold increased risk of incident HF than those without CKD [37]. The presence of HF in patients with CKD is associated with increased risk of death, more frequent hospitalizations, and reduced health-related quality of life [37–39]. The coexistence of HF (with a reduced or preserved ejection fraction [EF]) and CKD can manifest as cardiorenal syndrome (CRS), although comparatively less is known about CRS in HFpEF than in HFrEF [40]. CKD is commonly found in patients with HFpEF [41, 42], with a reported prevalence of 50–60% [43–45]. Similarly, the prevalence of HFpEF in patients with CKD is around 55% [36]. Risk factors and comorbidities common to both conditions include diabetes, hypertension, and obesity; T2DM in particular is a mediator and amplifier of the CRS process [46]. The pathophysiological mechanisms linking HFpEF and CKD have been described in detail [41, 42, 47, 48] and are presented in Fig. 1 [41]. Briefly, cardiac changes associated with HFpEF in patients with CKD include myocardial fibrosis, left ventricular (LV) hypertrophy, and diastolic dysfunction. Increased arterial stiffness, which is common to HFpEF and CKD, causes increased pulse pressure and pulse wave velocity that may be transmitted to the microvasculature. Pulmonary hypertension (resulting from LV dysfunction and metabolic injury in metabolic syndrome) is common in patients with HFpEF and leads to increased central venous pressure and systemic congestion, with subsequent decreased glomerular capillary blood flow and increased renal interstitial/tubular pressure. Importantly, comorbidities associated with HFpEF (such as diabetes and obesity) contribute to a state of systemic inflammation that induces oxidative stress in the coronary endothelium and causes metabolic changes that result in myocardial stiffness and interstitial fibrosis [49]. Equally, CKD is associated with various pathophysiologic consequences that increase LV workload and promote hypertrophy, including impaired sodium handling/volume overload, anemia, neurohormonal activation (renin-aldosterone-angiotensin system, and sympathetic nervous system), and disturbances to the vitamin-D-parathyroid hormone-fibroblast growth factor 23-Klotho axis. CKD also promotes a pro-inflammatory state that further contributes to oxidative stress, endothelial dysfunction, and vascular smooth muscle proliferation. CRS is defined as a disorder of the heart and kidneys, whereby acute or chronic dysfunction in one organ may induce acute or chronic dysfunction of the other [50]. The classification of CRS is presented in Fig. 2 [51]. Pathophysiology, diagnosis, and therapy for CRS were largely elucidated in patients with HFrEF, with relatively little data obtained in the context of HFpEF [40, 52]. Patients with HFpEF are more likely to have chronic systemic comorbidities (such as hypertension, T2DM, and obesity), whereas HFrEF often occurs as a consequence of acute or chronic loss of cardiomyocytes (due to ischemia, myocarditis, valvular disease, etc.) (reviewed in [53]) [49]; thus, any ensuing kidney disease may reflect these differences in etiology.

## SGLT2is in the treatment of patients with HFpEF and CKD

SGLT2is have emerged as a landmark therapy to reduce the burden of HF (incident and recurrent hospitalizations) and progression of kidney disease in patients with and without T2DM [2, 54–57]. Although the exact cardiorenal protective mechanisms of SGLT2is have not been fully elucidated, potential mechanisms include improved glycemic control, weight loss, and blood pressure reduction; metabolic reprogramming to shift the heart and kidney from carbohydrate consumption to lipid and ketones utilization; optimization of ventricular loading via effects on diuresis, natriuresis, and vascular function; modulation of kidney hemodynamics to correct hyperfiltration, albuminuria, and hypoxia; correction of inflammation and oxidative stress, resulting in antifibrotic effects; modulation of mitochondrial function; and enhancement of autophagy [54].

Data from numerous RCTs with SGLT2is have demonstrated significant reductions in adverse CV and kidney outcomes in patient populations across the spectrum of LV function, with and without T2DM. These RCTs are summarized in Table 1 (patients with HFpEF) and Table 2 (patients with HFrEF or worsening/acute HF). RCTs to investigate the effects of SGLT2is in patients with CKD also reported beneficial outcomes on hospitalization for HF, as presented in Table 3. Trial acronyms used below are defined in the tables.

**Table 1:** RCTs evaluating SGLT2is in patients with HFpEF (with or without T2DM)

Trial acronym	DELIVER	EMPEROR-Preserved	PRESERVED-HF	DETERMINE-Preserved	EMPERIAL-Preserved
Trial name	Dapagliflozin evaluation to improve the lives of patients with preserved ejection fraction heart failure	Empagliflozin outcome trial in patients with chronic heart failure with preserved ejection fraction	Dapagliflozin in preserved ejection fraction heart failure	Dapagliflozin effect on exercise capacity using a 6-minute walk test in patients with heart failure with preserved ejection fraction	Effect of empagliflozin on exercise ability and HF symptoms in patients with chronic heart failure
Therapeutic area	HFpEF	HFpEF	HFpEF	HFpEF	HFpEF
Clinicaltrials.gov identifier	NCT03619213	NCT03057951	NCT03030235	NCT03877224	NCT03448406
ClinicalTrials.gov URL	https://clinicaltrials.gov/ct2/show/NCT03619213	https://clinicaltrials.gov/ct2/show/NCT03057951	https://clinicaltrials.gov/ct2/show/NCT03030235	https://clinicaltrials.gov/ct2/show/NCT03877224	https://clinicaltrials.gov/ct2/show/record/NCT03448406
Trial completion	Mar 2022	Apr 2021	Aug 2021	Jul 2020	Oct 2019
Publication	Solomon et al., N Engl J Med 2022 [59] McCausland et al., JAMA Cardiol 2022 [62]	Anker et al., N Engl J Med 2021 [61]	Nassif et al., Nat Med 2021 [72]	Unpublished; details from clinicaltrials.gov	Abraham et al., Eur Heart J 2021 [73]
Intervention (once daily)	Dapagliflozin 10 mg vs. PBO	Empagliflozin 10 mg vs. PBO	Dapagliflozin 10 mg vs. PBO	Dapagliflozin 10 mg vs. PBO	Empagliflozin 10 mg vs. PBO
LVEF eligibility	> 40%	> 40%	≥ 45%	> 40%	> 40%
eGFR exclusion (mL/min/1.73 m^2^)	< 25	< 20 or requiring dialysis	< 20	< 25	< 20 or requiring dialysis
Population	Randomized = 6263 (dapagliflozin, 3131; PBO, 3132)	Randomized = 5988 (empagliflozin, 2997; PBO, 2991)	Randomized = 324 (dapagliflozin, 162; PBO, 162)	Randomized = 504 (dapagliflozin, 253; PBO, 251)	Randomized = 315 (empagliflozin, 157; PBO, 158)
Trial duration	Median 2.3 years	Median 26.2 months	12 weeks	16 weeks	12 weeks
Primary endpoint	Composite of worsening HF (unplanned HHF, or urgent HF visit) or CV death	Composite of CV death or HHF	Change from baseline to week 12 in KCCQ-CSS	Change from baseline to week 16 in KCCQ-TSS, KCCQ-PLS, and 6MWTD	Change from baseline to week 12 in 6MWTD
Primary endpoint achieved?	Yes	Yes	Yes	No	No
Details	18% reduced risk of primary outcome with dapagliflozin (16.4% vs. 19.5% in PBO group; HR: 0.82, 95% CI 0.73–0.92; p < 0.001) Baseline kidney function did not affect primary composite CV outcome (eGFR ≥ 60 mL/min/1.73 m^2^: HR: 0.84, 95% CI 0.70–1.00; eGFR 45– < 60 mL/min/1.73 m^2^: HR: 0.68; 95% CI 0.54–0.87; eGFR < 45 mL/min/1.73 m^2^: HR: 0.93, 95% CI 0.76–1.14; p for interaction = 0.16)	21% reduced risk of primary outcome with empagliflozin (13.8% vs. 17.1% in PBO group; HR: 0.79, 95% CI 0.69–0.90; p < 0.001)	Mean 12-week change in KCCQ-CS was 5.8 points (95% CI 2.3–9.2; p = 0.001) in favor of dapagliflozin	Dapagliflozin had no effect on the primary outcome measures; median difference dapagliflozin vs. PBO 3.16 (95% CI 0.36 to 6.01; p = 0.08) in KCCQ-TSS, 3.12 (95% CI –0.09 to 5.37; p = 0.23) in KCCQ-PLS, and 1.6 m (95% CI –5.9 to 9.0; p = 0.67) in 6MWTD	Empagliflozin had no effect on the primary outcome; median difference at week 12 empagliflozin vs. PBO 4.0 m (95% CI –5.0 to 13.0; p = 0.37) in 6MWTD
Kidney endpoint	Exploratory analysis: effect of treatment on eGFR slope Post hoc analysis: (1) composite of first sustained ≥ 50% decline in eGFR from baseline; (2) development of ESKD (from AE reporting or sustained decline in eGFR < 15 mL/min/1.73 m^2^); or (3) death due to kidney causes	Secondary endpoint: rate of decline from baseline in eGFR Other prespecified endpoint: composite of chronic dialysis or renal transplant or sustained reduction from baselines in eGFR of ≥ 40% (< 15 mL/min/1.73 m^2^ for patients with baseline eGFR ≥ 30 mL/min/1.73 m^2^, or < 10 mL/min/1.73 m^2^ for patients with baseline eGFR < 30 mL/min/1.73 m^2^) (defined per clinicaltrials.gov entry)	No kidney endpoints included	No kidney endpoints included	No kidney endpoints included
Kidney endpoint achieved?	Yes (exploratory endpoint only)	Yes (secondary endpoint only)	Not applicable	Not applicable	Not applicable
Details	Dapagliflozin slowed rate of eGFR decline (from baseline to month 36: difference, + 0.5 [95% CI 0.1–0.9] mL/min/1.73 m^2^ per year; p = 0.01; and from months 1–36: difference, + 1.4 [95% CI 1.0–1.8] mL/min/1.73 m^2^ per year; p < 0.001) Dapagliflozin had no effect on frequency of composite kidney outcome: dapagliflozin 2.5% vs. PBO 2.3% (HR: 1.08, 95% CI 0.79–1.49)	Empagliflozin slowed eGFR rate of decline vs. PBO (–1.25 vs. –2.62 mL/min/1.73 m^2^ per year [difference, 1.36]; (95% CI 1.06–1.66; p < 0.001) Empagliflozin had no effect on frequency of composite kidney outcome: empagliflozin 3.6% vs. PBO 3.7% (HR: 0.95, 95% CI 0.73–1.24)			

*6MWTD* 6-min walk test distance

*AE* adverse event, *CI* confidence interval, *CSS* Clinical Summary Score, *CV* cardiovascular, *EF* ejection fraction, *eGFR* estimated glomerular filtration rate, *ESKD* end-stage kidney disease, *HbA1C* hemoglobin A1C, *HF* heart failure, *HFpEF* heart failure with preserved ejection fraction, *HHF* hospitalization for heart failure, *HR* hazard ratio, *KCCQ* Kansas City Cardiomyopathy Questionnaire, *LVEF* left ventricular ejection fraction, *P* probability, *PBO* placebo, *PLS* Physical Level Score, *RCT* randomized controlled trial, *SGLT2i* sodium-glucose cotransporter-2 inhibitor, *T2DM* type 2 diabetes mellitus, *TSS* Total Symptom Score

**Table 2:** RCTs evaluating SGLT2is in patients with HFrEF or worsening/acute HF (with or without T2DM)

Trial acronym	DAPA-HF	EMPEROR-Reduced	SOLOIST-WHF	**CHIEF-HF**	**EMPULSE**
Trial name	Dapagliflozin and prevention of adverse outcomes in heart failure	Empagliflozin outcome trial in patients with chronic heart failure with reduced ejection fraction	Effect of sotagliflozin on cardiovascular events in participants with type 2 diabetes post worsening heart failure	A study on impact of canagliflozin on health status, quality of life, and functional status in heart failure	Empagliflozin in patients hospitalized with acute heart failure who have been stabilized
Therapeutic area	HFrEF	HFrEF	HF (inpatient)	HF	HF (inpatient)
ClinicalTrials.gov identifier	NCT03036124	NCT03057977	NCT03521934	NCT04252287	NCT04157751
ClinicalTrials.gov URL	https://clinicaltrials.gov/ct2/show/record/NCT03036124	https://clinicaltrials.gov/show/NCT03057977	https://clinicaltrials.gov/ct2/show/record/NCT03521934	https://clinicaltrials.gov/ct2/show/NCT04252287	https://clinicaltrials.gov/ct2/show/record/NCT04157751
Trial completion	Jul 2019	May 2020	Jun 2020 (terminated due loss of sponsor funding)	Nov 2021	Jun 2021
Publication	McMurray et al., N Engl J Med 2019 [5] Jhund et al., Circl 2021 [102]	Packer et al., N Engl J Med 2020 [68] Zannad et al., Circl 2021 [92]	Bhatt et al., N Engl J Med 2021 [71]	Spertus et al., Nat Med 2022 [74]	Voors et al., Nat Med 2022 [75]
Intervention (once daily)	Dapagliflozin 10 mg vs. PBO	Empagliflozin 10 mg vs. PBO	Sotagliflozin 200–400 mg vs. PBO	Canagliflozin 100 mg vs. PBO	Empagliflozin 10 mg vs. PBO
LVEF eligibility	≤ 40%	≤ 40%	None (inpatient admission due to worsening HF regardless of EF)	None (> 40% and ≤ 40% eligible)	None (inpatient admission with acute HF regardless of EF)
eGFR exclusion (mL/min/1.73 m^2^)	< 30	< 20 or requiring dialysis	< 30	< 30 or requiring dialysis	< 20 or requiring dialysis
Population	Randomized = 4744 (dapagliflozin, 2373; PBO, 2371)	Randomized = 3730 (empagliflozin, 1863; PBO, 1867)	Randomized = 1222 (sotagliflozin, 608; PBO, 614)	Randomized = 476 (ITT = 448: canagliflozin, 222; PBO, 226)	Randomized = 530 (empagliflozin, 265; PBO, 265)
Trial duration	Median 18.2 months	Median 16 months	Median 9 months	12 weeks	90 days
Primary endpoint	Composite of worsening HF or CV death	Composite of CV death or HHF	Total number of CV deaths and hospitalizations and urgent HF visits	Change from baseline to week 12 in KCCQ-TSS	Hierarchical composite of death, HF events, time to first HF event, or ≥ 5-point change from baseline in KCCQ-TSS at 90 days
Primary endpoint achieved?	Yes	Yes	Yes	Yes	Yes
Details	26% reduced risk of primary outcome with dapagliflozin (16.3% vs. 21.2% in PBO group; HR: 0.74, 95% CI 0.65–0.85; p < 0.001)	25% reduced risk of primary outcome with empagliflozin (19.4% vs. 24.7% in PBO group; HR: 0.75, 95% CI 0.65–0.86; p < 0.001)	33% reduced risk of primary outcome (events per 100 PY) with sotagliflozin (51.0 vs. 76.3 in PBO group; HR: 0.67, 95% CI 0.52–0.85; p < 0.001)	Mean 12-week change in KCCQ-TSS at week 12 was 4.3 points (95% CI 0.8–7.8; p = 0.016) in favor of canagliflozin	Win ratio 1.36 in favor of empagliflozin (53.89% wins vs. 39.71% wins in PBO group; 95% CI 1.09–1.68; p = 0.0054)
Kidney endpoint	Secondary endpoint: Worsening kidney function (composite of sustained decline in eGFR ≥ 50%, ESKD [sustained eGFR < 15 mL/min/1.73 m^2^, long-term dialysis, or kidney transplantation], or renal death)	Secondary endpoint: Rate of decline from baseline in eGFR Other prespecified endpoint: Composite of chronic dialysis or renal transplant or sustained reduction from baseline in eGFR of ≥ 40%, or a sustained eGFR < 15 mL/min/1.73 m^2^ for patients with baseline eGFR ≥ 30 mL/min/1.73 m^2^, or a sustained eGFR < 10 mL/min/1.73 m^2^ for patients with baseline eGFR < 30 mL/min/1.73 m^2^	Secondary endpoint (revised): Change in eGFR during follow-up	No kidney endpoints included	Occurrence of chronic dialysis or renal transplant or sustained reduction in eGFR from baseline ≥ 40% (eGFR < 15 mL/min/1.73 m^2^ for patients with baseline eGFR ≥ 30 mL/min/1.73 m^2^, sustained eGFR < 10 mL/min/1.73 m^2^ for patients with baseline eGFR < 30 mL/min/1.73 m^2^)
Kidney endpoint achieved?	Composite-no; slowed eGFR decline-yes	Yes	No	Not applicable	No
Details	Dapagliflozin 1.2% vs. PBO 1.6% (HR: 0.71, 95% CI 0.44–1.16; p = 0.17) From days 14–720: dapagliflozin –1.09 (95% CI –1.40 to −0.77) vs. PBO –2.85 (95% CI –3.17 to −2.53) mL/min/1.73 m^2^ per year; p < 0.001	Empagliflozin slowed eGFR rate of decline vs. PBO (–0.55 vs.–2.28 mL/min/1.73 m^2^ per year [difference, 1.73]; 95% CI 1.10–2.37; p < 0.001) Empagliflozin reduced the frequency of the composite renal outcome: empagliflozin 1.6% vs. PBO 3.1% (HR: 0.50, 95% CI 0.32–0.77)	Change in eGFR favored PBO; sotagliflozin –0.34 vs. PBO –0.18 mL/min/1.73 m^2^ (difference –0.16; 95% CI –1.30 to 0.98; p value NA, as not included in hierarchical-testing strategy)		Not incorporated into model

*CI* confidence interval, *CV* cardiovascular, *EF* ejection fraction, *eGFR* estimated glomerular filtration rate, *ESKD* end-stage kidney disease, *HbA1C* hemoglobin A1C, *HF* heart failure, *HFrEF* heart failure with reduced ejection fraction, *HHF* hospitalization for heart failure, *HR* hazard ratio, *KCCQ* Kansas City Cardiomyopathy Questionnaire, *NA* not applicable, *P* probability, *PBO* placebo, *PY* patient-years, *RCT* randomized controlled trial, *SGLT2i* sodium-glucose cotransporter-2 inhibitor, *TSS* Total Symptom Score

**Table 3:** RCTs evaluating SGLT2is in patients with CKD (with or without T2DM)

Trial acronym	EMPA-KIDNEY	SCORED	CREDENCE	DAPA-CKD
Trial name	Study of heart and kidney protection with empagliflozin	Effect of sotagliflozin on cardiovascular and renal events in participants with type 2 Diabetes and Moderate renal impairment who are at cardiovascular risk	Canagliflozin and renal events in diabetes with established nephropathy Clinical Evaluation	Dapagliflozin and prevention of adverse outcomes in chronic kidney disease
Therapeutic area	CKD (HF, 10% patients; T2DM, 44% patients; HF + T2DM, 14% patients)	CKD (HF, 31% patients; EF, ≥ 50% 16% patients)	CKD (HF, ~ 15% patients)	CKD (HF, ~ 11% patients; T2DM, 67%)
ClinicalTrials.gov identifier	NCT03594110	NCT03315143	NCT02065791	NCT03036150
ClinicalTrials.gov URL	https://clinicaltrials.gov/ct2/show/record/NCT03594110	https://clinicaltrials.gov/ct2/show/NCT03315143	https://clinicaltrials.gov/ct2/show/NCT02065791	https://clinicaltrials.gov/ct2/show/NCT03036150
Trial completion	Jan 2025 (primary outcome completion Jul 2022)	Jul 2020 (terminated due loss of sponsor funding)	Oct 2018	Jun 2020
Publication	EMPA-KIDNEY Collaborative Group, N Engl J Med 2022 [80] EMPA-KIDNEY Collaborative Group, Nephrol Dial Transplant 2022 [79]	Bhatt et al., N Engl J Med 2021 [76]	Perkovic et al., N Engl J Med 2019 [77]	Heerspink et al., N Engl J Med 2020 [78]
Intervention (once daily)	Empagliflozin 10 mg vs. PBO	Sotagliflozin 200–400 mg vs. PBO	Canagliflozin 100 mg vs. PBO	Dapagliflozin 10 mg vs. PBO
eGFR inclusion (mL/min/1.73 m^2^)	≥ 20 to < 45 or ≥ 45 to < 90 with UACR ≥ 200 mg/g (or protein:creatinine ≥ 300 mg/g)	≥ 25 to ≤ 60	≥ 30 and < 90	≥ 25 to ≤ 75
Other relevant inclusion criteria	None	T2DM, HbA1c ≥ 7%	T2DM, HbA1c ≥ 6.5% and ≤ 12.0% UACR > 300 mg/g and ≤ 5000 mg/g Aged ≥ 30 years	With/without T2DM UACR ≥ 200 to ≤ 5000 mg/g
Population	Randomized = 6609 (empagliflozin, 3304; PBO, 3305)	Randomized = 10,584 (sotagliflozin, 5292; PBO, 5292)	Randomized = 4401 (canagliflozin, 2202; PBO, 2199)	Randomized = 4304 (dapagliflozin, 2152; PBO, 2152)
Trial duration	Median 2.0 years	Median 16 months	Median 2.6 years (trial stopped early due to clear benefit observed for primary outcome)	Median 2.4 years
Primary endpoint	Composite of kidney disease progression (ESKD, sustained decline in eGFR to < 10 mL/min/1.73m^2^, sustained decline of ≥ 40% in eGFR from randomization, or renal death) or CV death	Total number of CV deaths, HF hospitalizations, and urgent HF visits	Composite of doubling of serum creatinine, ESKD, renal or CV death	Composite of ≥ 50% decrease in eGFR, ESKD, renal or CV death
Primary endpoint achieved?	Yes	Yes	Yes	Yes
Details	28% reduced risk of primary outcome with empagliflozin (13.1% vs. 16.9% in PBO group; HR: 0.72; 95% CI 0.64–0.82; p < 0.001)	26% reduced risk of primary outcome (events per 100 PY) with sotagliflozin (5.6 vs. 7.5 in PBO group; HR: 0.74, 95% CI 0.63–0.88; p < 0.001)	30% reduced risk of primary outcome (events per 1000 PY) with canagliflozin (43.2 vs. 61.2 in PBO group; HR: 0.70, 95% CI 0.59–0.82; p < 0.00001)	39% reduced risk of primary outcome with dapagliflozin (9.2% vs. 14.5% in PBO group; HR: 0.61, 95% CI 0.51–0.72; p < 0.001)
Other kidney endpoint	See primary endpoint	Secondary endpoint (revised): composite of first occurrence of sustained decrease ≥ 50% in eGFR from baseline for ≥ 30 days, long-term dialysis, renal transplantation, or sustained eGFR of < 15 mL/min/1.73 m^2^ for ≥ 30 days	Secondary endpoint: composite of ESKD, doubling of serum creatinine, or renal death	Secondary endpoint: composite of ≥ 50% decrease in eGFR, ESKD, or renal death
Other kidney endpoint achieved?	See primary endpoint	No	Yes	Yes
Details		Composite endpoint (events per 100 PY): Sotagliflozin 0.5 vs. PBO 0.7 (HR: 0.71, 95% CI 0.46–1.08; p value NA, as not included in hierarchical-testing strategy)	34% reduced risk of composite endpoint (events per 1000 PY) with canagliflozin (27.0 vs. 40.4 in PBO group; HR: 0.66, 95% CI 0.53–0.81; p < 0.001)	44% reduced risk of composite endpoint with dapagliflozin (6.6% vs. 11.3% in PBO group; HR: 0.56, 95% CI 0.45–0.68; p < 0.001)
HF endpoint	Secondary endpoint: composite of HHF or CV death	Secondary endpoint: total number HHF and urgent HF visits	Secondary endpoint: composite of HHF or CV death	Secondary endpoint: composite of HHF or CV death
HF endpoint achieved?	No	Yes	Yes	Yes
Details	Composite endpoint: empagliflozin 4.0% vs. PBO 4.6% (HR: 0.84, 95% CI 0.67–1.07; p = 0.15)	33% reduced risk of endpoint (events per 100 PY) with sotagliflozin (3.5 vs. 5.1 in PBO group; HR: 0.67, 95% CI 0.55–0.82; p < 0.001)	31% reduced risk of endpoint (events per 1000 PY) with canagliflozin (31.5 vs. 45.4 in PBO group; HR: 0.69, 95% CI 0.57–0.83; p < 0.001)	29% reduced risk of composite endpoint with dapagliflozin (4.6% vs. 6.4% in PBO group; HR: 0.71, 95% CI 0.55–0.92; p < 0.009)

*CI* confidence interval, *CKD* chronic kidney disease, *CV* cardiovascular, *EF* ejection fraction, *eGFR* estimated glomerular filtration rate, *ESKD* end-stage kidney disease, *HF* heart failure, *HHF* hospitalization for heart failure, *HR* hazard ratio, *P* probability, *PBO* placebo, *PY* patient-years, *RCT* randomized controlled trial, *SGLT2i* sodium-glucose cotransporter-2 inhibitor, *T2DM* type 2 diabetes mellitus, *UACR* urine albumin-creatinine ratio

### HF trials

The DELIVER [58, 59] and EMPEROR-Preserved [60, 61] trials demonstrated that SGLT2i therapy reduced the composite endpoint of HF events or CV death in patients with HFpEF (EF > 40%) compared with placebo. Baseline kidney function did not influence the effect of dapagliflozin on the primary outcome in DELIVER [62]. Exploratory analysis showed that dapagliflozin slowed the rate of eGFR decline over time [62], as did empagliflozin in EMPEROR-Preserved [61], which was consistent with the preservation of kidney function demonstrated in prior studies of these and other SGLT2is [63]. However, use and interpretation of the eGFR slope is debated [64], and it may not predict intrinsically the kidney composite outcome in clinical trials investigating HF and SGLT2i therapy. A prespecified composite kidney endpoint was included in EMPEROR-Preserved but not in DELIVER, where it was analyzed post hoc [65]. In EMPEROR-Preserved, the prespecified composite kidney outcome gave a neutral finding [63], with a reduction of 5% for empagliflozin versus placebo that was not statistically significant [61]. A similar trend was observed in DELIVER [62]. When kidney outcomes data from EMPEROR-Preserved were reanalyzed using more conventional endpoints employed in large-scale SGLT2i trials and a meta-analysis (≥ 50% decrease in eGFR, and including renal death [66]), the hazard ratio (HR) for the effect of empagliflozin on major kidney outcomes was 0.78 (95% confidence interval [CI] 0.54–1.13) [66]. Furthermore, the effect size of empagliflozin on kidney outcomes (≥ 50% decrease in eGFR and renal death) was influenced by LVEF (P-trend = 0.02), with higher LVEFs showing reduced rates of protection [66]: for LVEF 41–49%, HR was 0.41 (95% CI 0.20–0.85), whereas for LVEF ≥ 60%, HR was 1.24 (95% CI 0.66–2.33) [66]. This followed the trend observed with empagliflozin for LVEF and HF hospitalization, where an attenuated effect occurred at LVEF ≥ 60% [67]. The effect of LVEF was confirmed by a planned pooled analysis of data from EMPEROR-Preserved and its sister trial in patients with HFrEF, EMPEROR-Reduced [68] [69], in which the reduction in cumulative annual deficit in eGFR for empagliflozin vs, placebo was higher in patients with HFrEF than HFpEF (EMPEROR-Reduced 1.77 [95% CI 0.80–2.74] mL/min/1.73 m^2^ vs. EMPEROR-Preserved 0.94 [95% CI 0.60–1.27] mL/min/1.73 m^2^) [4]. One explanation was that some patients enrolled into EMPEROR-Preserved had atrial fibrillation and not HFpEF, as around 50% of all trial participants were noted to have atrial fibrillation at baseline [61], particularly those with LVEF ≥ 60–65% [63]. Another suggestion was that the original data simply reflected the initial decline in kidney function observed with SGLT2is, despite the eventual protective effects on the kidney in the longer term [63].

A recent prespecified meta-analysis of DELIVER and EMPEROR-Preserved (N = 12,251) reported that SGLT2is reduced the composite endpoint of CV death or first hospitalization for HF (HR: 0.80, 95% CI 0.73–0.87), with consistent results across both endpoint components (CV death: HR: 0.88, 95% CI 0.77–1.00; first hospitalization for HF: HR: 0.74, 95% CI 0.67–0.83) [70]. Although safety could not be compared directly between these trials due to differences in the determination and reporting of adverse events, serious adverse events in both trials were numerically less frequent in the SGLT2i treatment groups versus the placebo groups [70]. This meta-analysis included a post hoc analysis of three further RCTs with SGLT2is, including patients with HFrEF (DAPA-HF [5] and EMPEROR-Reduced [68]) and those with worsening HF requiring inpatient care (SOLOIST-WHF [71]) [70]. When all five RCTs were considered (N = 21,947), the combined meta-analysis showed SGLT2is reduced the risk of the primary composite endpoint, its components, and all-cause mortality; namely, composite CV death or hospitalization for HF (HR: 0.77, 95% CI 0.72–0.82), CV death (HR: 0.87, 95% CI 0.79–0.95), first hospitalization for HF (HR: 0.72, 95% CI 0.67–0.78), and all-cause mortality (HR: 0.92, 95% CI 0.86–0.99) [70]. Treatment effects were consistent across LVEF subgroups (and the other subgroups examined).

Other RCTs with SGLT2is in patients with HFpEF (namely, PRESERVED-HF, DETERMINE-Preserved, and EMPERIAL-Preserved) evaluated improvement in HF symptoms via health-related quality of life scores (Kansas City Cardiomyopathy Questionnaire [KCCQ]-Clinical Summary Score [CSS], -Total Symptom Score [TSS], and -Physical Limitation Score [PLS]) and exercise capacity [6-min walk test distance (6MWTD)]. PRESERVED-HF reported significant improvements in KCCQ and 6MWTD with dapagliflozin vs. placebo [72]; however, these findings were not replicated in DETERMINE-Preserved, in which dapagliflozin had no effect on outcomes (data unpublished). Similarly, EMPERIAL-Preserved reported there was no effect of empagliflozin on improving exercise capacity via 6MWTD [73]. Health-related quality of life scores were also used to assess SGLT2is in patients with HF (not limited to pEF) in two further RCTs: CHIEF-HF [74] and EMPULSE [75]. CHIEF-HF evaluated the effect of canagliflozin on HF symptoms using health-related quality of life scores for KCCQ-TSS, but the trial did not include kidney outcomes. Canagliflozin was associated with a beneficial change in KCCQ-TSS vs. placebo over 12 weeks [74]. EMPULSE assessed the effect of empagliflozin in patients with acute HF who had been stabilized in the hospital. A win ratio in favor of empagliflozin vs. placebo was reported for the hierarchical composite primary endpoint (death, HF events, and KCCQ-TSS) [75]. A secondary kidney endpoint was included but was not incorporated into the statistical model.

### CKD trials with an HF endpoint

Several RCTs with SGLT2is in patients with CKD included an HF outcome. SCORED [76], CREDENCE [77], and DAPA-CKD [78] demonstrated that sotagliflozin, canagliflozin, and dapagliflozin, respectively, reduced the risk of the primary kidney and CV endpoints when compared with placebo and achieved the secondary endpoints in which HF was a component. SCORED and CREDENCE included patients with T2DM, whereas patients with or without T2DM were enrolled into DAPA-CKD. EMPA-KIDNEY [79, 80] included a range of patients with CKD who were at risk of disease progression. Comorbid conditions at baseline included T2DM (44% of patients), HF (10%), and T2DM plus HF (14%); there was a wide representation of eGFR values (≥ 20 to < 90 mL/min per 1.73 m^2^), 34% of patients had an eGFR < 30 mL/min per 1.73 m^2^, and 48% had urine albumin-creatinine ratio (UACR) < 300 mg/g; also, the primary renal diagnosis contained several non-diabetes–related glomerular diseases, including IgA nephropathy (12% of patients), focal segmental glomerulosclerosis (3%), and membranous nephropathy (1%) [79]. Empagliflozin therapy led to a lower risk of the primary outcome (kidney disease progression or CV death) than placebo, and results were consistent among patients with or without diabetes (predominantly T2DM) and across subgroups defined by eGFR ranges [80]. However, there was heterogeneity for the primary outcome across UACR strata, with most of the effect being driven by patients with UACR > 300 mg/g (HR: 0.67, 95% CI 0.58–0.78) [80]. No significant between-group differences were observed with respect to the composite secondary outcome of hospitalization for HF or CV death (empagliflozin 4.0% versus placebo 4.6%) or death from any cause (4.5% and 5.1%, respectively) [80].

One of the notable omissions in the major SGLT2i trials in HF is the minimal reporting on UACR. Even if the kidney outcomes relating to eGFR-based definitions do not align at this time, the addition of UACR and time-based changes (an accepted surrogate for CKD progression) may provide useful information on the overall kidney effects of these agents in HF.

### Possible heterogeneity of HFpEF trial populations

**Fig. 3:**
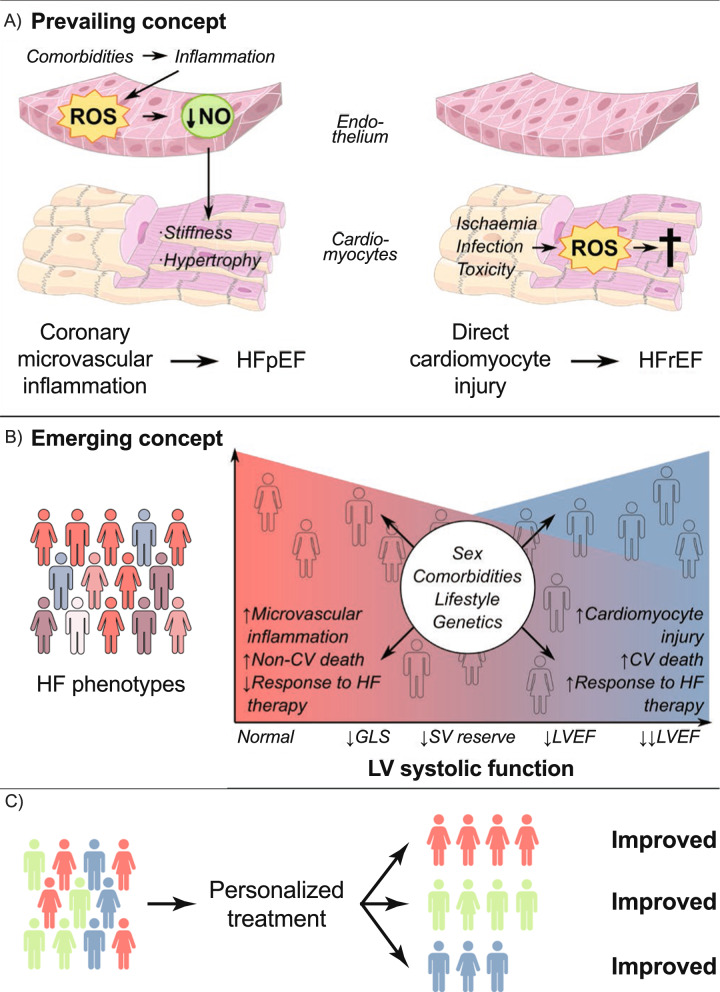
Evolution of pathophysiological understanding of HFpEF [84]. **A** Prevailing concept of HFpEF and HFrEF as separate diseases, HFpEF is caused by microvascular inflammation and HFrEF is caused by cardiomyocyte loss. **B** Emerging concept of heart failure as phenotypes overlapping across the spectrum of LV systolic function. There is a gradual change in underlying pathophysiology, mode of death, and response to HF therapies across the LVEF spectrum, with influences from genetics, sex, comorbidities, and lifestyle. **C** Personalized treatment of HFpEF. Different phenotypes (based on clinical, imaging, biomarker and/or transcriptomic data) are represented by red, green and blue colors. Personalized treatment: considering the phenotype-specific response to medical therapy, a targeted approach using specific drugs in specific phenotypes could lead to net clinical benefit for all patients. *CV* cardiovascular, *GLS* global longitudinal strain, *HF* heart failure, *HFpEF* heart failure with preserved ejection fraction, *HFrEF* heart failure with reduced ejection fraction, *LV* left ventricle, *LVEF* left ventricular ejection fraction, *NO* nitric oxide, *ROS* reactive oxygen species, *SV* stroke volume. Reproduced from Heart 2022, volume 108, pages 1342–1350, Gevaert AB, Kataria R, Zannad F, Sauer AJ, Damman K, Sharma K, et al. Heart failure with preserved ejection fraction: recent concepts in diagnosis, mechanisms and management, Copyright 2022, with permission from BMJ Publishing Group Ltd

The apparent attenuation of empagliflozin’s effect at higher LVEF levels in EMPEROR-Preserved has provoked questions regarding the effectiveness of SGLT2is across the spectrum of EFs in HF [81]. However, it has become evident that HFpEF is not a single entity, and patients with HFpEF are a heterogeneous group with a range of contributory conditions, such as atrial fibrillation, hypertensive heart disease, obesity, cardiac hypertrophy, and myocardial fibrosis (reviewed in [81]). Several HFpEF phenotypes have been identified via machine learning algorithms or hierarchical clustering [12, 82, 83] and are linked to differences in outcomes [83]; these include younger people with mild HF, people with diabetes and obesity, those with atrial fibrillation and CKD, men with atrial fibrillation, and frail older women with atrial fibrillation (reviewed in [12]). There is now an emerging concept of HF as a spectrum of LV systolic function containing various overlapping phenotypes (reviewed in [84] and presented in Fig. 3A and B) [84]. Some of these groups may be expected to be more responsive to SGLT2i treatment than others [81], such as patient phenotypes related to obesity and metabolism/inflammation [85, 86]. In addition, other less common cardiac disorders may present with a HFpEF phenotype. These are broadly divided into conditions affecting the myocardium (such as inherited or acquired infiltrative, restrictive, inflammatory, or genetic cardiomyopathies) and those altering cardiac loading conditions (such as hypertension, congenital or acquired valvular and structural defects, rhythm abnormalities, etc.) [87]. Some of these presentations may not be responsive to SGLT2i treatment, particularly those caused by infiltrative diseases, such as cardiac amyloidosis [88]. Cardiac amyloidosis causes restrictive cardiomyopathy, of which the major clinical presentation is HFpEF, with symptoms caused by raised LV filling pressure secondary to increased stiffness and reduced elasticity of the heart tissue [89]. Kidney involvement may occur as part of the primary condition (e.g., systemic amyloidosis is a cause of type 5 CRS) or may be secondary to the ensuing cardiac disease. The importance of recognizing patients with previously undiagnosed cardiac amyloidosis in clinical trials for HFpEF was described recently, and this condition may contribute further to the heterogeneity of HFpEF populations and the failure to obtain positive results in some studies [90].

Factors related to the design of EMPEROR-Preserved may also be relevant to the observed effect of LVEF. For example, the inclusion of patients with LVEF > 40%, which encompasses HFmrEF (LVEF 41–49%), and the use of a variety of imaging techniques to measure LVEF up to 6 months before study entry, which increases variability and may underestimate potential changes over time [81]. However, the DELIVER trial design may refute this, as it also enrolled patients with LVEF > 40% and documented the EF over a longer period prior to trial enrollment (≤ 12 months), with assessment only via echocardiography or cardiac magnetic resonance imaging [58].

### Secondary kidney outcomes in HFpEF vs. HFrEF

A further question is why the secondary kidney outcomes with HFpEF look different when compared with HFrEF. In a comparison of EMPEROR-Reduced and DAPA-HF [91], in which patients with HFrEF were enrolled, both trials reported similar and significant effects of SGLT2is in reducing the decline in the eGFR slope (mean slope of eGFR change vs. placebo: 1.73 and 1.78 mL/min/1.73 m^2^ per year for empagliflozin and dapagliflozin, respectively) [91]. However, the composite kidney outcome showed a statistical decrease in EMPEROR-Reduced (0.50; 95% CI 0.32–0.77) but not in DAPA-HF (0.71, 95% CI 0.44–1.16), possibly due to fewer kidney events in the latter because of a higher eGFR entry criterion (≥ 20 vs. ≥ 30 mL/min/1.73 m^2^, respectively) and differences in the eGFR decline defined in the composite kidney outcome (≥ 40% for EMPEROR-Reduced vs. ≥ 50% decline in DAPA-HF) [91]. A prespecified analysis of EMPEROR-Reduced, in which patients were categorized by the presence or absence of CKD at baseline (defined as eGFR < 60 mL/min/1.73 m^2^ or UACR > 300 mg/g), investigated the direct impact on kidney events via a prespecified composite kidney outcome (defined as a sustained profound decline in eGFR, chronic dialysis, or transplant) [92]. Empagliflozin reduced the slope of eGFR decline in patients with CKD (1.11 [95% CI 0.23–1.98] mL/min/1.73 m^2^ per year) and without CKD (2.41 [95% CI 1.49–3.32] mL/min/1.73 m^2^ per year), and the risk of the composite kidney outcome was similarly reduced in patients with and without CKD (HR: 0.53, 95% CI 0.31–0.91 vs. HR: 0.46, 95% CI 0.22–0.99, respectively) [92].

Despite these analyses, the reason why the kidney outcomes appear to be less impressive in patients with HFpEF largely remains unknown. The comparison of EMPEROR-Reduced and DAPA-HF presents relevant points concerning the level of eGFR at trial entry, number of kidney events, and differences in definitions, but it is equally possible that the difference in kidney outcomes is simply due to chance. Furthermore, it may be erroneous to postulate that HFrEF and HFpEF consistently behave differently with respect to kidney outcomes. Data from a post hoc analysis of renal outcomes in the Prospective Comparison of angiotensin receptor-neprilysin inhibitor (ARNI) with angiotensin receptor blocker (ARB) Global Outcomes in HF with Preserved Ejection Fraction trial (PARAGON-HF; NCT01920711) demonstrated a significant reduction in the prespecified kidney composite outcome (time to first occurrence of either ≥ 50% reduction in eGFR, ESKD, or death from renal causes) [93], even though the primary outcome (composite of total HF hospitalizations and CV death) was not achieved [43]. Conversely, the United Kingdom Heart and Renal Protection-III trial (UK HARP-III; ISRCTN11958993) showed no benefits in the kidney with ARNI use in patients with CKD only [94], although the results may have been affected by the trial design (patient characteristics, heterogeneous CKD etiologies, small study size, short follow-up duration, etc. [93]). These data highlight the need for obtaining a more accurate phenotype for patients with HF and CKD (carried out using methods other than cut-offs for eGFR and LVEF) to better determine which patients will respond to different guideline-directed medical therapies (as depicted in Fig. 3B).

## Emerging therapies for HFpEF and CKD: finerenone

Although SGLT2is are undoubtedly valuable in the management of HF and CKD, other drugs are also important. Finerenone is one of the standards of care in patients with DKD [95, 96]. It is a selective, non-steroidal mineralocorticoid receptor (MR) antagonist (MRA) that blocks MR-mediated sodium reabsorption and MR overactivation and has demonstrated anti-inflammatory and anti-fibrotic effects in preclinical models of kidney and CV disease [97]. The complementary phase 3 RCTs, Finerenone in Reducing Kidney Failure and Disease Progression in Diabetic Kidney Disease (FIDELIO-DKD; NCT02540993 [98]) and Finerenone in Reducing Cardiovascular Mortality and Morbidity in Diabetic Kidney Disease (FIGARO-DKD; NCT02545049 [99]) comprise the largest cardiorenal outcomes program in CKD in T2DM to date [100]. Patients with chronic symptomatic HFrEF were excluded from the FIDELIO and FIGARO trials. FIDELITY was a prespecified pooled analysis of efficacy and safety data from FIDELIO and FIGARO and allowed for evaluation across the range of CKD severity [96] (N = 13,026; broad spectrum of CKD and T2DM; all patients were treated with an optimized dose of angiotensin-converting enzyme inhibitor or ARB) [100]. Approximately 8% of all trial participants were noted to have HF at baseline. FIDELITY provided evidence of both CV and renal protection with finerenone compared with placebo. The analysis showed a 14% risk reduction in the composite CV outcome (consisting of CV death, non-fatal myocardial infarction [MI], non-fatal stroke, or hospitalization for HF) for finerenone vs. placebo (12.7% vs. 14.4%, respectively; HR: 0.86 [95% CI 0.78–0.95]; p = 0.0018) and 23% reduction in risk of the composite kidney outcome (consisting of sustained ≥ 57% decrease in eGFR from baseline over ≥ 4 weeks or renal death) for finerenone vs. placebo (5.5% vs. 7.1%; HR: 0.77 [95% CI 0.67–0.88]; p = 0.0002) [96, 100]. Hospitalization for HF was the primary contributor to the CV benefit observed in the FIDELITY analysis, with a 22% risk reduction (HR: 0.78, 95% CI 0.66–0.92; p = 0.0030) [100]. Per the US Food and Drug Administration, finerenone is now indicated to reduce the risk of sustained eGFR decline, ESKD, CV death, non-fatal MI, and hospitalization for HF in adults with CKD associated with T2DM [101].

## Recommendations for management of HFpEF

**Fig. 4:**
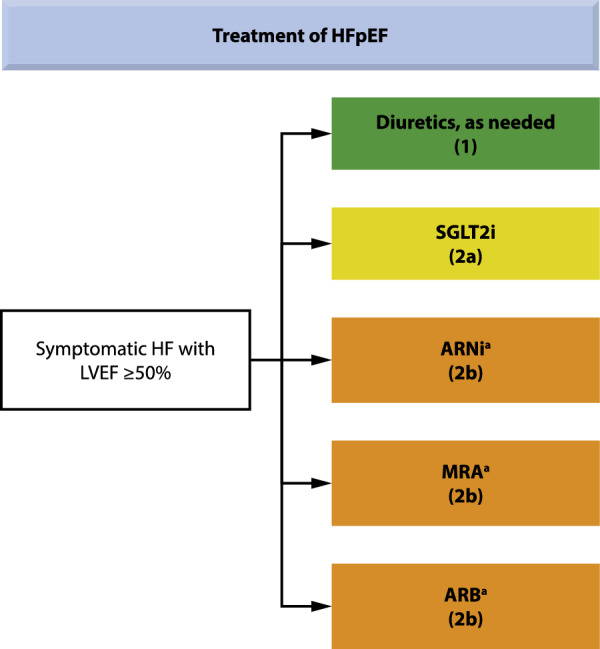
Recommendations for patients with preserved LVEF (≥ 50%), per AHA/ACC/HFSA 2022 [8]. Medication recommendations for HFpEF are displayed. *ARB* angiotensin receptor blocker, *ARNI* angiotensin receptor-neprilysin inhibitor, *HF* heart failure, *HFpEF* heart failure with preserved ejection fraction, *LVEF* left ventricular ejection fraction, *MRA* mineralocorticoid receptor antagonist, *SGLT2i* sodium-glucose cotransporter-2 inhibitor. *Greater benefit in patients with LVEF closer to 50%. Reproduced from J Am Coll Cardiol. 2022, volume 79(17), pages e263–e421, Heidenreich PA, Bozkurt B, Aguilar D, et al. 2022 AHA/ACC/HFSA guideline for the management of heart failure: a report of the American College of Cardiology/American Heart Association Joint Committee on Clinical Practice Guidelines, Copyright (2022), with permission from The American Heart Association, Inc., The American College of Cardiology Foundation, and The Heart Failure Society of America

Joint guidelines from the American Heart Association/American College of Cardiology/Heart Failure Society of America (AHA/ACC/HFSA 2022) now include the use of SGLT2is for patients with HFpEF (Class of Recommendation 2a, evidence moderate; benefit >  > risk) [8] due to their benefits in decreasing HF hospitalizations and CV mortality [61] (presented in Fig. 4). However, these recommendations were issued before the results of DELIVER were published, and will likely be updated when the new data are taken into consideration. MRAs and ARNIs may also be considered for decreasing hospitalizations in selected patients with HFpEF, particularly those at the lower end of the LVEF spectrum, per the AHA/ACC/HFSA guidelines (Class of Recommendation 2b, evidence weak; benefit ≥ risk) [8]. European Society of Cardiology (ESC) guidelines were published in September 2021 prior to the availability of data from recently completed trials with SGLT2is in HFpEF; thus, no recommendations regarding disease-modifying therapies are provided. However, the use of SGLT2is (dapagliflozin and empagliflozin) is recommended by the ESC in patients with HFrEF to reduce the risk of hospitalization for HF and death [7].

## Limitations

There are several limitations associated with this work. Only two databases were used in the search strategy. As the retrieved references from the searches were only assessed by one reviewer, there is a possibility of selection bias.

## Conclusions

SGLT2is have demonstrated efficacy and safety in treating HFpEF in patients with comorbid CKD, with and without T2DM. The efficacy of SGLT2is appears to be a class effect. Data from some clinical trials have led clinicians to question whether SGLT2is are effective across the spectrum of EFs in HF, and whether there may be a difference in kidney outcomes between patients with HFpEF vs. HFrEF. Further analysis of the individual trial designs and participant characteristics reveal potentially mitigating factors that may explain the relevant sets of ostensibly neutral results and highlights the need to obtain more accurate phenotypes for patients with HF and CKD (using more nuanced methods than cut-off values for eGFR and LVEF) to better determine which patients might respond to different guideline-directed medical therapies. Furthermore, due to their high risk of developing HFpEF, patients with CKD may benefit from therapies such as SGLT2is, ARNis, ARBs, and MRAs even if they have not yet been diagnosed with HF.

## Data Availability

Data sharing is not applicable to this article, as no new data were created or analyzed in this report.

## Abbreviations

6MWTD: 6-Minute walk test distance
ACC: American College of Cardiology
ACR: Albumin-creatinine ratio
AHA: American Heart Association
ARB: Angiotensin receptor blocker
ARNI: Angiotensin receptor-neprilysin inhibitor
ASCVD: Atherosclerotic cardiovascular disease
CI: Confidence interval
CKD: Chronic kidney disease
CRS: Cardiorenal syndrome
CV: Cardiovascular
CVD: Cardiovascular disease
CSS: Clinical Summary Score
DALY: Disability-adjusted life year
DKD: Diabetes kidney disease
EF: Ejection fraction
eGFR: Estimated glomerular filtration rate
ESC: European Society of Cardiology
ESKD: End-stage kidney disease
GFR: Glomerular filtration rate
HF: Heart failure
HFmrEF: Heart failure with mildly reduced ejection fraction
HFpEF: Heart failure with preserved ejection fraction
HFrEF: Heart failure with reduced ejection fraction
HFSA: Heart Failure Society of America
HR: Hazard ratio
KCCQ: Kansas City Cardiomyopathy Questionnaire
LV: Left ventricular
LVEF: Left ventricular ejection fraction
MI: Myocardial infarction
MR: Mineralocorticoid receptor
MRA: Mineralocorticoid receptor antagonist
RCT: Randomized controlled trial
SGLT2: Sodium-glucose cotransporter-2
SGLT2i: Sodium-glucose cotransporter-2 inhibitor
T2DM: Type 2 diabetes mellitus
TSS: Total Summary Score
UACR: Urine albumin-creatinine ratio
US: United States
